# Supplementary material for: Citizenship, Migration and Mobility in a Pandemic (CMMP): A global dataset of COVID-19 restrictions on human movement
Source: PLoS One. 2021 Mar 9;16(3):e0248066. doi: 10.1371/journal.pone.0248066 (PMC7943018; doi:10.1371/journal.pone.0248066)
Supplement: S1 Codebook — (DOCX) [file pone.0248066.s001.docx]

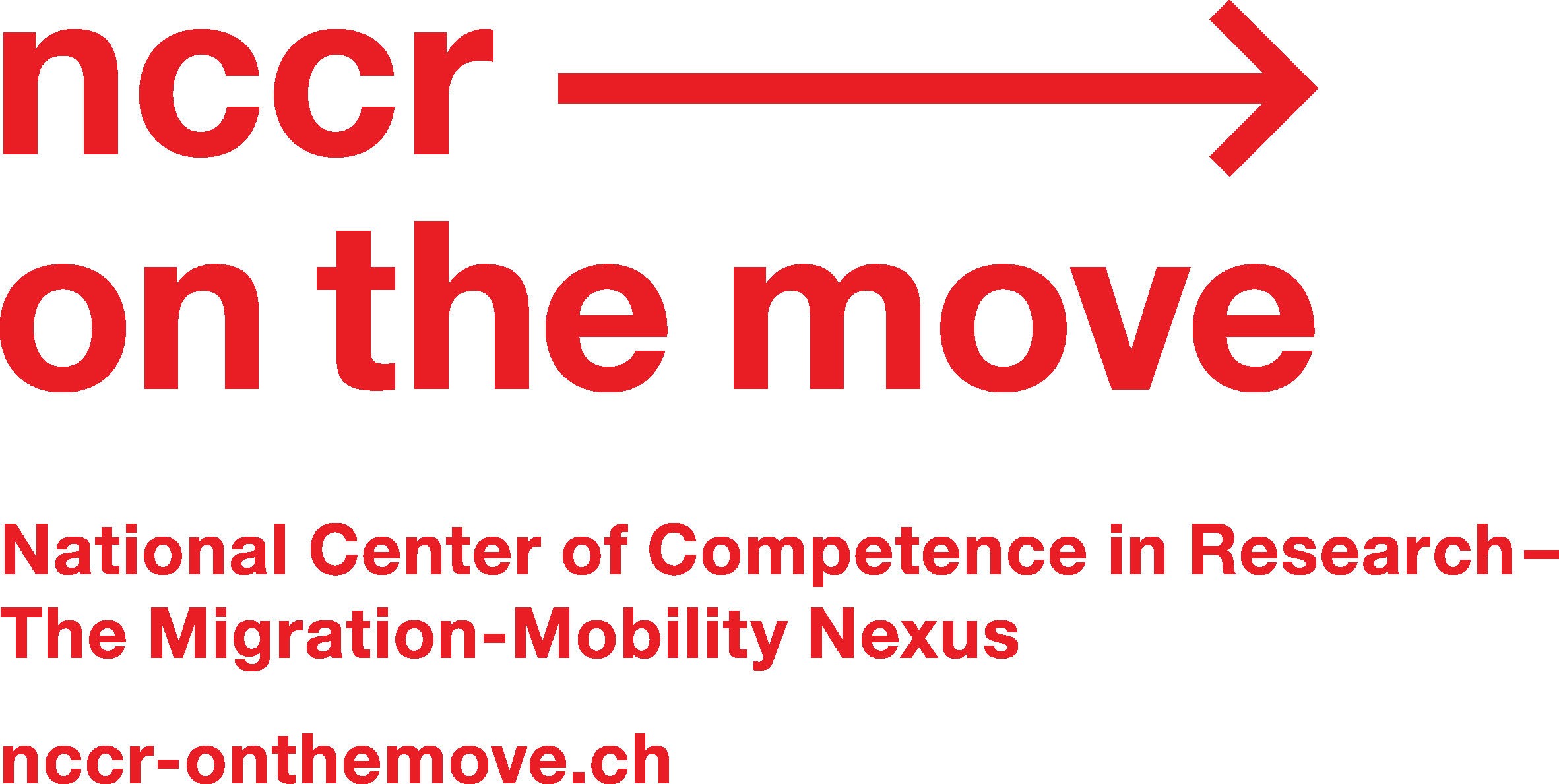


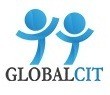


International Travel Restrictions in Response to the COVID-19 Outbreak

Codebook, Description of Microdata

26 August 2020


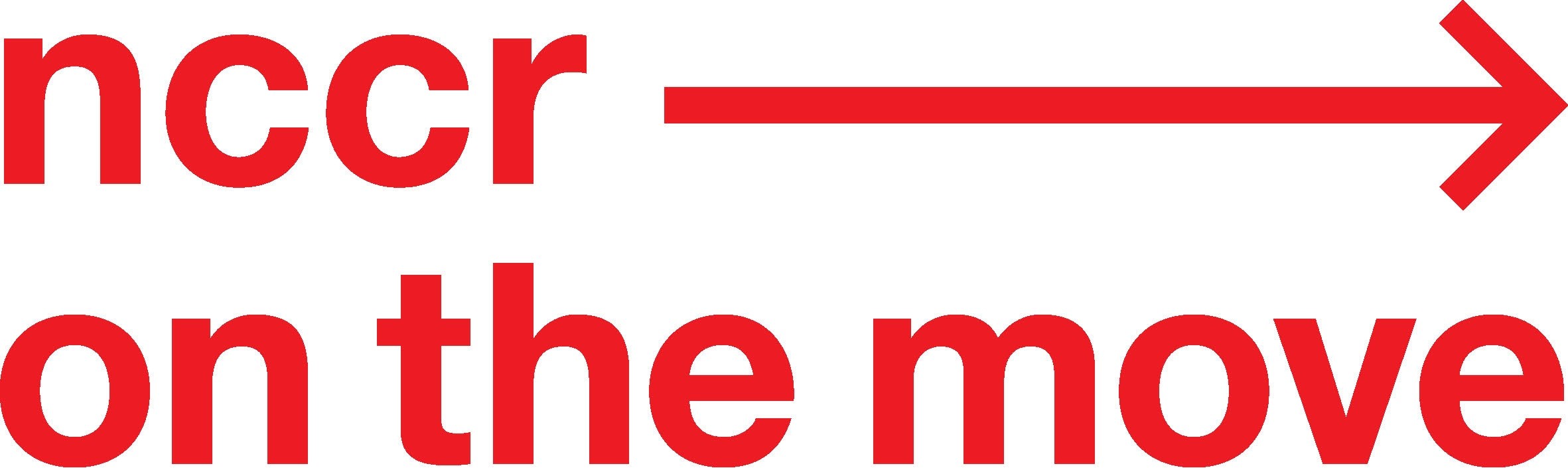

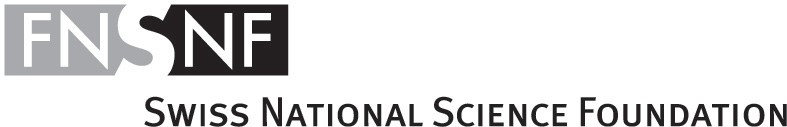

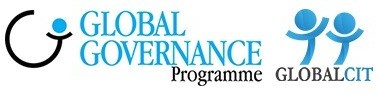


# Impressum

### Concept and production

**Production Date** 26 August 2020

This document was generated using the IHSN Microdata Management Toolkit Additional design and coding by Andreas Perret

#
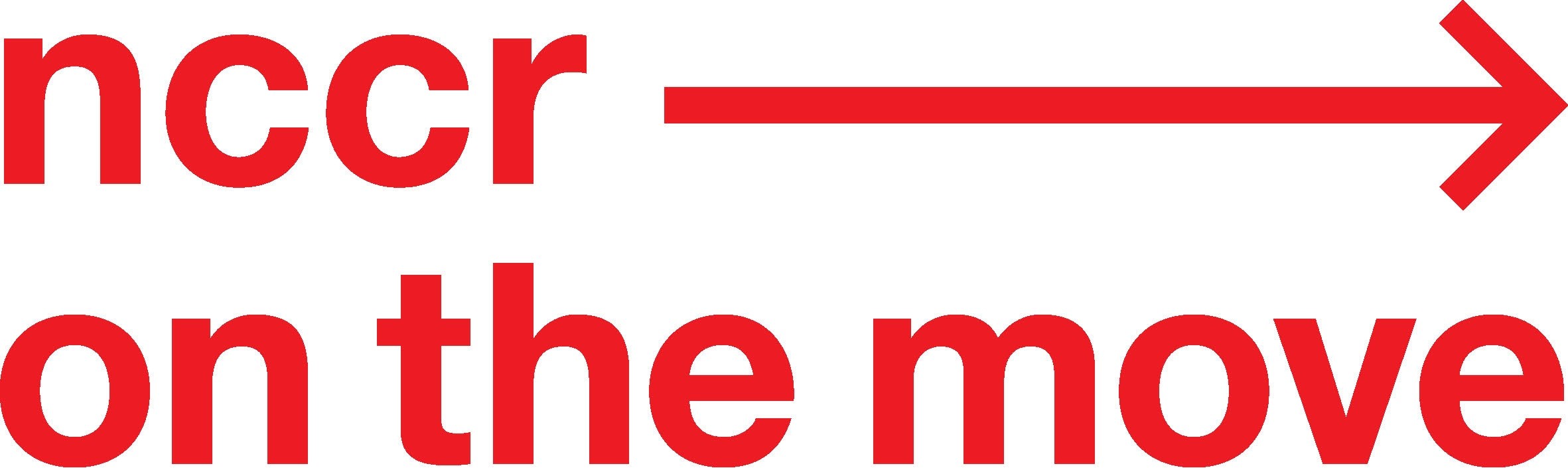

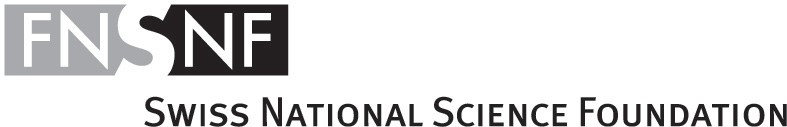

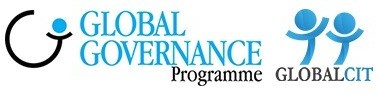
Table of Contents

International Travel Restrictions in Response to the COVID-19 Outbreak (CMMP- B). 1

Overview. 1

Scope. 1

Coverage. 1

Sampling. 1

Data Collection. 2

Accessibility. 2

Rights & Disclaimer 2

[Files Description. 3](#_TOC_250005)

Covid-19 Travel Restrictions 3

Countries. 3

Targets. 3

[Variables Groups. 4](#_TOC_250004)

[Variables List 5](#_TOC_250003)

Covid-19 Travel Restrictions 5

Countries. 5

Targets. 5

[Alphabetical List 6](#_TOC_250002)

[Variables Description 7](#_TOC_250001)

Covid-19 Travel Restrictions 7

Countries. 9

Targets. 9

[Appendices. 12](#_TOC_250000)

International Travel Restrictions in Response to the COVID-19 Outbreak (CMMP-B)

**2020**

Overview

#### **Abstract** The dataset features systematic information on border closures in response to the COVID-19 outbreak from 1 March 2020 to 31 May 2020. It documents the evolution of the types and scope of international travel restrictions and exceptions to them in global coverage.

**Version of the microdata**

Original data compiled from the "Mobility Impacts" documents of the International Migration Organization (IOM), government websites, newspaper agencies.

Notes: This is an ongoing study at the time of the publication of this dataset. Please bear in mind that future versions may differ from the descriptions included in this codebook.

Notes

International travel restrictions in response to the COVID-19 outbreak is an ongoing study at the time of the publication of this dataset.

Please bear in mind that future versions may differ from the descriptions included in this codebook.

**State of data** 1 June 2020

#### **Type** Administrative Records, Other [ad/oth]

**Series** This dataset is used for the second part of the study "Citizenship, Migration and Mobility in a Pandemic" (CMMP-B)

**Kind of Data** Administrative records data [adm]

**Persistent Identifier**

#### TravelRestrictions

Scope

**Scope** We track restrictions on human movement in the context of the COVID-19 outbreak from 1 March 2020 on.

Coverage

**Universe** Countries for which the research team found travel restrictions based on data from the International Migration Organization (IOM), government websites, newspaper agencies.

**Unit of Analysis** Individual travel restrictions and exceptions, by emitting country and restricted country.

**Geographic Coverage**

Global


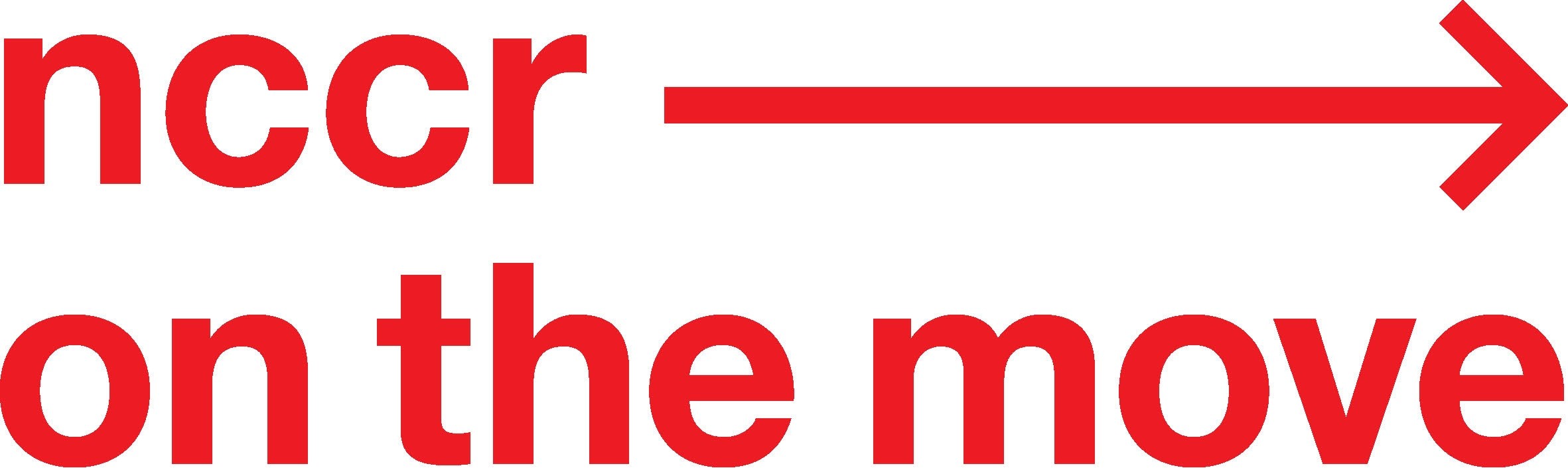

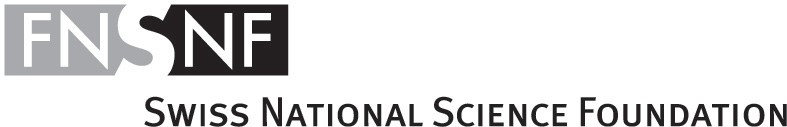

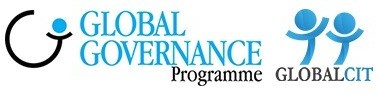


Sampling

**Sampling Procedure**

**Deviations from Sample Design**

The data lists all countries that issue travel restrictions based on data from the International Migration Organization (IOM), government websites, newspaper aaggeenncciieess...

All original data is collected in the dataset. For clarity of display on visualizations, restricting or restricted countries can be omitted through the use of excluding variables.

Data Collection

**Data Collection Dates**

**Data Collection Mode**

**Data Collection Notes**

**Data Collector(s) Other Processing**

start 2020-03-01

end 2020-05-31

Internet [int]

This data collection is ongoing.

Data coded by XY in separate spreadsheets for each day. The resulting file was then converted and restructured in this dataset by XY.

Additional controls were conducted by XY and XY.

Validity checks were conducted by a team of research assistants.

These files are the basic information used for visualisation of the data found under the following link: <https://tabsoft.co/3fxs9d0>

Accessibility

**Access Authority** XY

**Contact(s)** XY

**Access Conditions** Data freely available under CC BY ND

### Citation Requirements

Rights & Disclaimer

#### **Disclaimer** This is an ongoing collation project of live data. If you see any inaccuracies in the underlying data or want to provide specific feedback on the analysis or another aspect of the project please contact us at XY.

# Files Description

Dataset contains 3 file(s)

Covid-19 Travel Restrictions

**File Content** The file describes the restrictions to international travel registered on miscellaneous websites announcing travel restrictions. The indicators capture the type of restriction (self-isolation, quarantine, screening, medical certificate, visa, no entry), the target (nationality, presence, residence), and exceptions (family, government authorization, humanitarian protection, humanitarian workers, military, nationals, visa-type, official delegations, partner countries, residents, risk-free individuals, transit, transport personnel, work-related travel, other specific exceptions) across countries and time. The types of restrictions are ranked by whether an individual is allowed to cross the border. The multiple primary key is based on combined variables "Date", "Country Number", "Type of restriction", "Restriction criteria" and "Target number"

**Cases** 4'412'690

### Variable(s) 5

**Producer** Andreas Perret

### Version of the microdata

#### In order to reduce the size of this file, only numerical values have been recorded. The corresponding values for issuing Countries are listed in the "Countries" dataset. The corresponding values for restricted countries and exceptions are listed in the "Target" dataset.

**Notes** CSV file containing one line per issuing country, type and base of restriction, possible exceptions and restricted country

Countries

**File Content** List of countries as recognized by the Swiss Federal Statistical Office. The primary key is based on the variable "Country Number"

**Cases** 258

### Variable(s) 2

**Producer** Swiss Federal Statistical Office

#### **File Content** List or areas subjected to travel restrictions or exceptions to the restrictions. This value is at the most granular level, some areas do not apply to whole countries but regions or cities, exceptions are recorded at the detailed levels. For values recoded in countries and exceptions in broad categories, use the variable "TargetGroup". The primary key is based on the variable "Target number"

Targets

**Cases** 822

**Variable(s)** 7

# Variables Groups

Dataset contains 0 groups

# Variables List

Dataset contains 14 variable(s)

File Covid-19 Travel Restrictions

| **Name** | | **Label** | **Data Type** | **Valid** |
| --- | --- | --- | --- | --- |
| 1 | **DateTxt** | Date | discrete | 4'412'690 |

| 2 | **SG_BFSNR** | Country Number | discrete | - |
| --- | --- | --- | --- | --- |
| 3 | **RestrictionNb** | Type of Restriction | discrete | 4'412'690 |
| 4 | **BasedonNB** | Restriction Criteria | discrete | 4'412'690 |
| 5 | **TargetNb** | Target Number | discrete | - |

File Countries

| **Name** | | **Label** | **Data Type** | **Valid** |
| --- | --- | --- | --- | --- |
| 1 | **SG_BFSNR** | Country Number | discrete | - |

2 **SG_NAMKE** Country name discrete 258

File Targets

| **Name** | | **Label** | **Data Type** | **Valid** |
| --- | --- | --- | --- | --- |
| 1 | **TargetNb** | Target Number | discrete | - |

| 2 | **QualifierNB** | Record qualifier (numeric value) | discrete | 822 |
| --- | --- | --- | --- | --- |
| 3 | **Qualifier** | Record qualifier | discrete | 822 |
| 4 | **TargetGroup** | Regrouped value | discrete | 822 |
| 5 | **Target** | Label of target | discrete | 822 |
| 6 | **Exclude** | Indication of exclusion | discrete | 822 |
| 7 | **SG_BFSNR** | - | discrete | 296 |

# Alphabetical List

#### Dataset contains 14 variable(s)

| **Name** | **Label** | **File** | **Page** |
| --- | --- | --- | --- |
|  |  |  |  |
| **BasedonNB** | Restriction Criteria | **Covid-19 Travel Restrictions** | **8** |
| **DateTxt** | Date | **Covid-19 Travel Restrictions** | **7** |
| **Exclude** | Indication of exclusion | **Targets** | **11** |
| **Qualifier** | Record qualifier | **Targets** | **9** |
| **QualifierNB** | Record qualifier (numeric value) | **Targets** | **9** |
| **RestrictionNb** | Type of Restriction | **Covid-19 Travel Restrictions** | **8** |
| **SG_BFSNR** | Country Number | **Covid-19 Travel Restrictions** | **7** |
| **SG_BFSNR** | Country Number | **Countries** | **9** |
| **SG_BFSNR** | - | **Targets** | **11** |
| **SG_NAMKE** | Country name | **Countries** | **9** |
| **Target** | Label of target | **Targets** | **11** |
| **TargetGroup** | Regrouped value | **Targets** | **10** |
| **TargetNb** | Target Number | **Covid-19 Travel Restrictions** | **9** |
| **TargetNb** | Target Number | **Targets** | **9** |

# Variables Description

Dataset contains 14 variable(s)

Comment: these figures indicate the number of cases found in the data file. They cannot be interpreted as summary statistics of the population of interest.

File Covid-19 Travel Restrictions

1 **DateTxt** Date

**Information** Data Type: discrete, Format: Character, Missing: *

**Statistics** Valid=4412690, Invalid=0

**Definition** Date when a given restriction enforced

*Frequency table not shown (92 Modalities)*

2 **SG_BFSNR** Country Number

**Information** Data Type: discrete, Format: numeric, Range: 8'100-8'683, Missing: *

**Definition** Country number, as defined by the Swiss Federal Statistical Office (see dataset "Countries" for labels) This value is provided in order to ease the use of this dataset with other sources.

## File Covid-19 Travel Restrictions

3 **RestrictionNb** Type of Restriction

**Information** Data Type: discrete, Format: numeric, Range: 1-6, Missing: *

**Statistics** Valid=4412690, Invalid=0

**Definition** Self-isolation

Individuals are allowed to enter the country in line with standard immigration policy and advised to self-isolate: they may be required to fill out a form saying where they intend to stay, and authorities have the right to make subsequent check on that address

Quarantine

Individuals are allowed to enter the country in line with standard immigration policy but are subject to mandatory quarantine in designed stations

Screening

Individuals are allowed to enter the country in line with standard immigration policy if they clear at the health control at the border, ranging from a simple measurement of the temperature to extensive symptom checks and swab sampling

Medical certificate

Individuals are allowed to enter the country in line with standard immigration policy if they are in possession of specific medical documents obtained prior to reaching the border point

Visa

Individuals are required to obtain entry visas that were not previously demanded, or existing visas suspended

No entry

Individuals are not allowed to enter the country

| Value | Label | N |  | Percentage |  |
| --- | --- | --- | --- | --- | --- |
| 1 | Medical certificate | 126'476 |  | 2.9% |  |
| 2 | No entry | 3'175'458 |  |  | 72.0% |
| 3 | Quarantine | 514'746 |  | 11.7% |  |
| 4 | Screening | 133'831 |  | 3.0% |  |
| 5 | Self-isolation | 276'886 |  | 6.3% |  |
| 6 | Visa restriction | 185'293 |  | 4.2% |  |

Comment: these figures indicate the number of cases found in the data file. They cannot be interpreted as summary statistics of the population of interest.

4 **BasedonNB** Restriction Criteria

**Information** Data Type: discrete, Format: numeric, Range: 1-3, Missing: *

**Statistics** Valid=4412690, Invalid=0

**Definition** Defines the criteria for restriction:

Nationality

The measure targets travelers who are nationals of one of the affected countries

Presence

The measure concerns travelers who have been physically present in one of the affected countries over the last 14 days

Residence

The measure targets travelers who legally reside in one of the affected countries

| Value | Label | N | Percentage | |
| --- | --- | --- | --- | --- |
| 1 | Nationality | 135'995 | | 3.1% |
| 2 | Presence | 4'232'973 |  | 95.9% |
| 3 | Residence | 43'722 | | 1.0% |

Comment: these figures indicate the number of cases found in the data file. They cannot be interpreted as summary statistics of the population of interest.

## File Covid-19 Travel Restrictions

5 **TargetNb** Target Number

**Information** Data Type: discrete, Format: numeric, Range: 1-829, Missing: *

**Definition** Points to a record in the "Targets" file that describes a country which is targeted by the restriction or an exception to a restriction.

## File Countries

1 **SG_BFSNR** Country Number

**Information** Data Type: discrete, Format: numeric, Range: 8'100-8'703, Missing: *

**Definition** Country number, as defined by the Swiss Federal Statistical Office

2 **SG_NAMKE** Country name

**Information** Data Type: discrete, Format: Character, Missing: *

**Statistics** Valid=258, Invalid=0

**Definition** Country label, as defined by the Swiss Federal Statistical Office

## File Targets

1 **TargetNb** Target Number

**Information** Data Type: discrete, Format: numeric, Range: 1-910, Missing: *

**Definition** Arbitrary number, key to the records in this file.

2 **QualifierNB** Record qualifier (numeric value)

**Information** Data Type: discrete, Format: numeric, Missing: *

**Statistics** Valid=822, Invalid=0

**Definition** This variable is provided to as a substitute to the string value of the Qualifier to speed-up of processes.

Value 1

2

Label

N Percentage 431

391

52.4%

47.6%

Comment: these figures indicate the number of cases found in the data file. They cannot be interpreted as summary statistics of the population of interest.

3 **Qualifier** Record qualifier

**Information** Data Type: discrete, Format: Character, Missing: *

**Statistics** Valid=822, Invalid=0

**Definition** Defines the record describes an area/country affected by a restriction, or an exception to a restriction.

Value Label Exception

Target

N Percentage 431

391

52.4%

47.6%

Comment: these figures indicate the number of cases found in the data file. They cannot be interpreted as summary statistics of the population of interest.

##
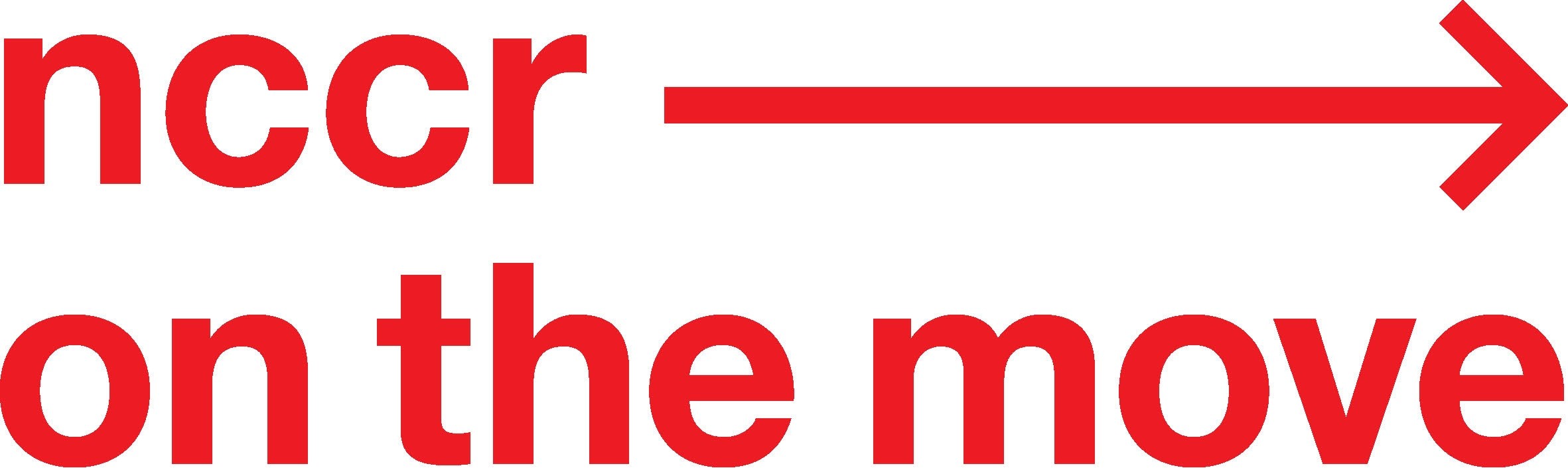

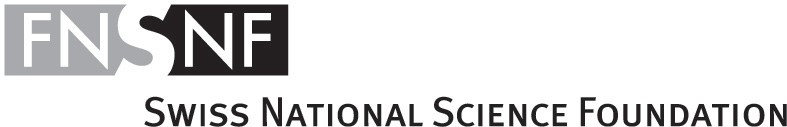

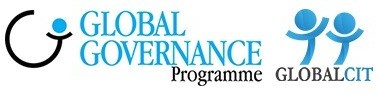
File Targets

4 **TargetGroup** Regrouped value

**Information** Data Type: discrete, Format: Character, Missing: *

**Statistics** Valid=822, Invalid=0

**Definition** Groupings of the "Target" values that simplify the data analysis.

Restricted areas are grouped into 212 countries. Exceptions are grouped into the following 15 categories:

Family of residents / nationals: Children/foster children/ward/spouse of a national or permanent resident; individuals unmarried and younger than 21 years who are brother or sister of a national or permanent resident

Government authorisation: Individuals with a valid letter of Prior Permission Granted from the government or from a consular office; those with landing privileges previously granted by the government

Humanitarian / aid workers: Healthcare, humanitarian, or elderly care professionals in the performance of their duties

Humanitarian protection: Protected person, under section 95.2 of the Canadian Immigration and Refugee Protection Act; refugees; persons who bring documentary reasons of force majeure or situation of need, or whose entry is permitted for humanitarian reasons; minors traveling without parents or legal tutor, pregnant women, elderly passengers or passengers with disabilities

Military personnel: Members of the army, United Nations interim Forces Nationals: Individuals who hold citizenship of the country

Long-term visa : Long-term visa holders, student pass holders, holders of employment in the country Official delegations: Accredited diplomats, government delegations - and their families

Other exception (specific): Individuals exercising visitation rights with a minor; travelers in an ongoing health treatment; individuals participating in a funeral; individuals travelling for health reasons; individuals visiting a critically or terminally ill family member; individuals who have a particular responsibility to care for residents or who have strong welfare reasons; individuals registered as an Indian under the Indian Act in Canada or registered with “The Palestinian Authority" passports and their spouses in Israel

Nationals of partner countries: Nationals and residents of areas with free movement agreements (e.g. countries belonging to the Countries belonging to the Schengen Area (Austria, Belgium, Czech Republic, Denmark, Estonia, Finland, France, Germany, Greece, Hungary, Iceland, Italy, Latvia, Liechtenstein, Lithuania, Luxembourg, Malta, Netherlands, Norway, Poland, Portugal, Slovakia, Slovenia, Spain, Sweden, and Switzerland)); nationals of neighboring countries; nationals of neighboring islands

Residents: Individuals who hold ID certifying residence in the country; social pass holders

Risk-free individuals: Individuals who submit a certificate for regulated quarantine or good health status issued by an agency authorised by the government

Transit: Individuals in transit

Transport personnel: Technical, directive personnel; cargo operators; airline crews; truck drivers

Work or business related travel: Individuals moving for professional purposes; seasonal workers; individuals with a cross-border commuter permit; individuals who can prove they are fulfilling commercial and contractual obligations

##
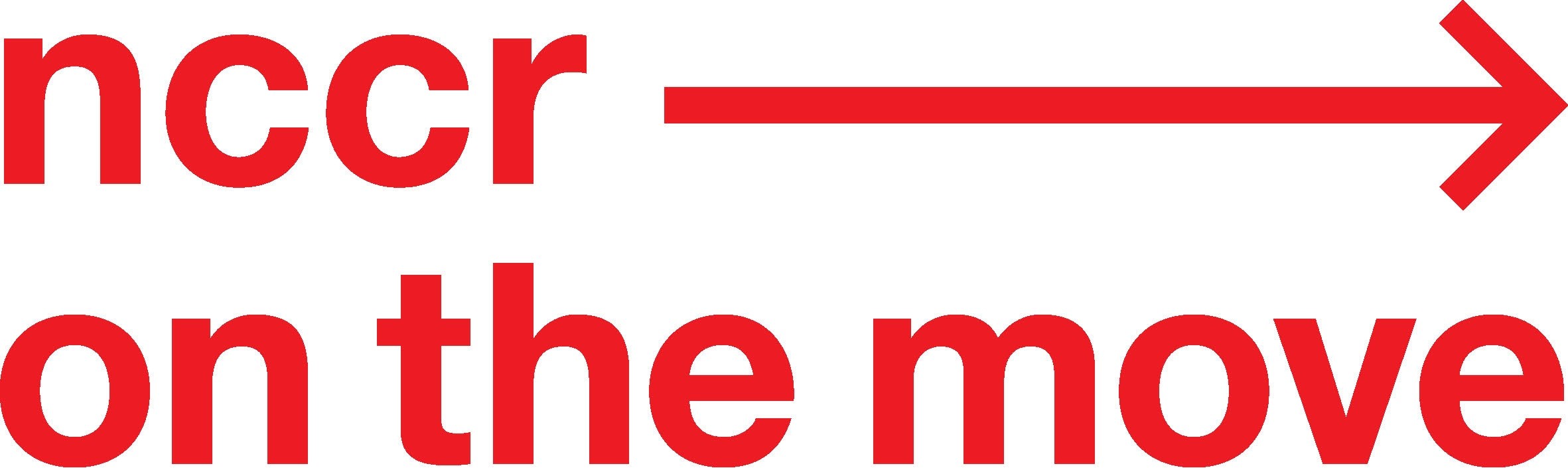

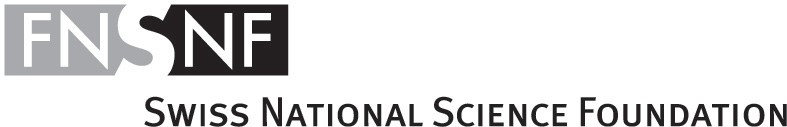

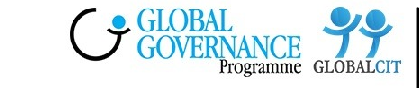
File Targets

5 **Target** Label of target

**Information** Data Type: discrete, Format: Character, Missing: *

**Statistics** Valid=822

**Definition** Text description of either an area (country, region, group of states) which is targeted by the restriction or an exception to a restriction.

6 **Exclude** Indication of exclusion

**Information** Data Type: discrete, Format: numeric, Range: 0-3, Missing: *

**Statistics** Valid=822, Invalid=0

**Definition** Indicates if the record should be omitted from analysis.

Applies only to restricting areas. 0 - value to be kept

1. - area excluded (for clarity of maps or because of obsolete territories)
2. - describes a broad area (such as "All countries) that should be excluded (these areas have been recoded in specific countries).

| Value | Label | N | Percentage | | |
| --- | --- | --- | --- | --- | --- |
| 0 | 727 | |  | | 88.4% |
| 1 | 83 | |  | 10.1% | |
| 2 | 12 | | 1.5% | | |

Comment: these figures indicate the number of cases found in the data file. They cannot be interpreted as summary statistics of the population of interest.

7 **SG_BFSNR**

**Information** Data Type: discrete, Format: numeric, Missing: *

**Statistics** Valid=296, Invalid=526

*Frequency table not shown (212 Modalities)*

#
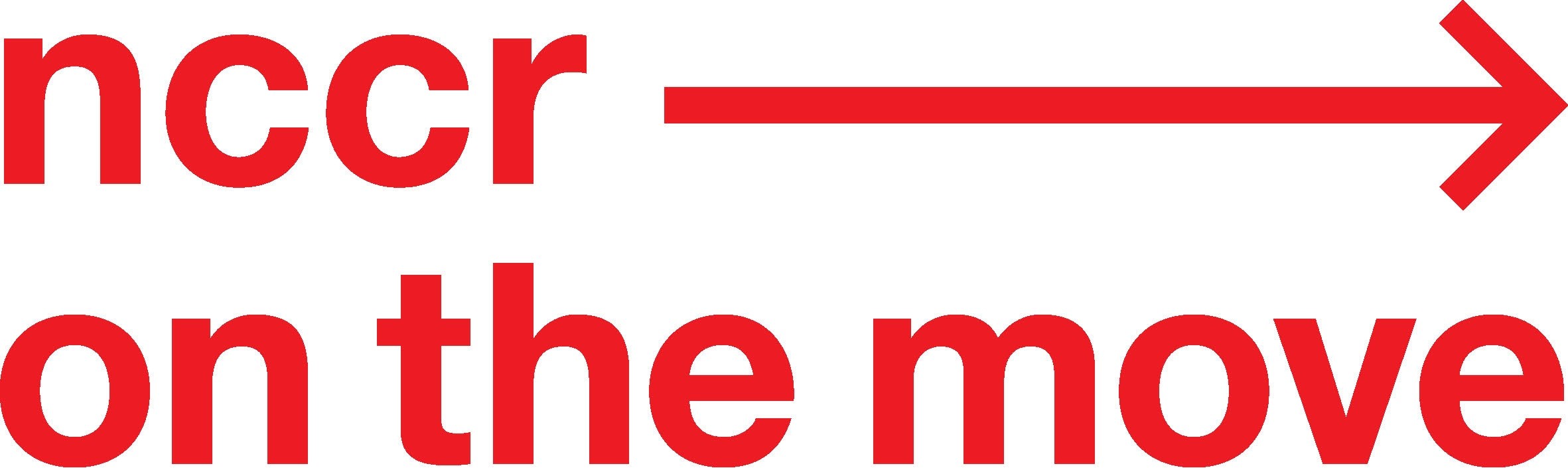

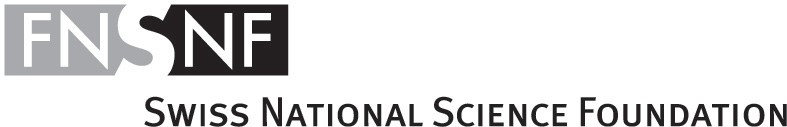

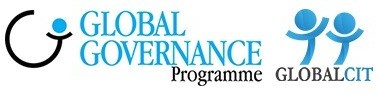
Appendices

## The documents listed below are available on the nccr - on the move website (nccr- onthemove.ch)

Other resources

*https://public.tableau.com/profile/nccr.on.the.move*

**Website**
