# Supplementary material for: Citizenship, Migration and Mobility in a Pandemic (CMMP): A global dataset of COVID-19 restrictions on human movement
Source: PLoS One. 2021 Mar 9;16(3):e0248066. doi: 10.1371/journal.pone.0248066 (PMC7943018; doi:10.1371/journal.pone.0248066)
Supplement: S2 Codebook — (DOCX) [file pone.0248066.s002.docx]

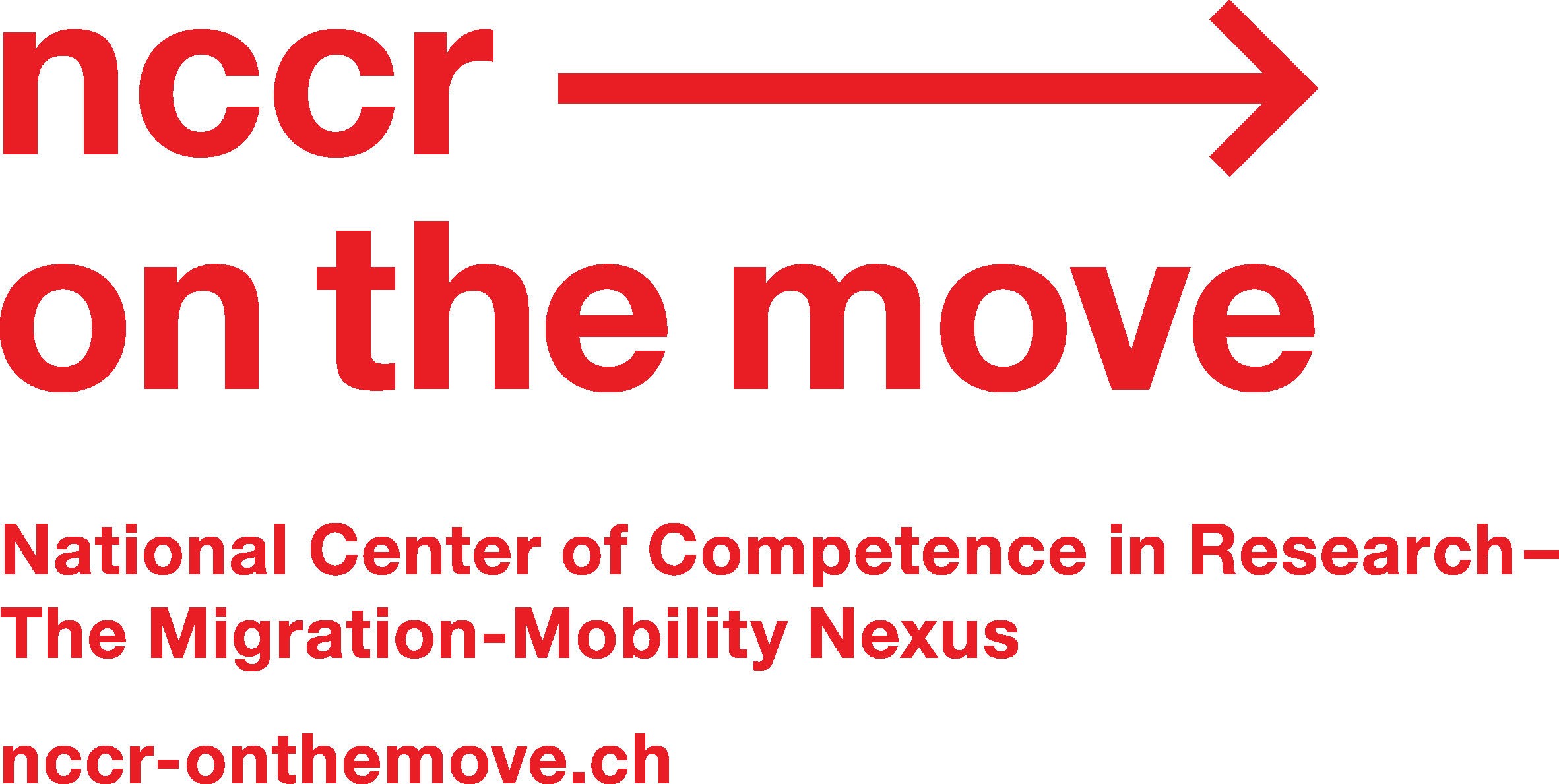


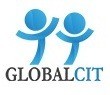


Mobility and Border Control in Response to the COVID-19 Outbreak

**Mobility restrictions across and within 27 European countries**

Codebook, Description of Microdata

26 August 2020

# Impressum

### Concept and production

**Production Date** 26 August 2020

This document was generated using the IHSN Microdata Management Toolkit Additional design and coding by Andreas Perret

# Table of Contents

Mobility and Border Control in Response to the COVID-19 Outbreak (CMMP- A). 1

Overview. 1

Scope. 1

Coverage. 1

Data Collection. 1

[Data Processing & Appraisal 2](#_TOC_250008)

[Accessibility. 2](#_TOC_250007)

[Files Description. 3](#_TOC_250006)

[Covid19 Mobility and Borders. 3](#_TOC_250005)

[Variables Groups. 4](#_TOC_250004)

[Variables List 5](#_TOC_250003)

Covid19 Mobility and Borders. 5

[Alphabetical List 6](#_TOC_250002)

[Variables Description 7](#_TOC_250001)

Covid19 Mobility and Borders. 7

[Appendices. 10](#_TOC_250000)

Mobility and Border Control in Response to the COVID-19 Outbreak (CMMP-A)

**Mobility restrictions across and within 27 European countries 2020**

Overview

**Abstract** The dataset Mobility and Border Control features systematic information on border closures and domestic lockdowns in response to the COVID-19 outbreak from 1 March to 31 May 2020.

### Version of the microdata

Original data collected from government websites and online newspapers. The data was gathered by the team of investigators and coded in a common master file.

**State of data** 1 June 2020

**Type** Administrative Records, Other [ad/oth]

**Series** This dataset is used for the first part of the study "Citizenship, Migration and Mobility in a Pandemic (CMMP)"

**Kind of Data** Administrative records data [adm]

### Persistent Identifier

CMMP-A

Scope

**Scope** We track restrictions on human movement in the context of the COVID-19 outbreak between 1 March 2020 and 31 May 2020.

Coverage

**Universe** 27 countries in Europe

**Unit of Analysis** Individual mobility restrictions at the borders and within 27 European countries.

### Geographic Coverage

27 countries

Data Collection

### Data Collection Dates

**Data Collection Mode**

**Data Collection Notes**

start 2020-03-01

end 2020-05-31

Internet [int]

This is an ongoing collation project of live data. If you see any inaccuracies in the underlying data or want to provide specific feedback on the analysis or another aspect of the project please contact us at XY

### Data Collector(s) XY

**Supervision** Data collection was organized by XY according to a structure defined by XY

## Data Processing & Appraisal

**Data Editing** Data coded by XY

**Other Processing** These files are the basic information used for visualisation of the data found under the following link:

https://tabsoft.co/2YqC5is

## Accessibility

### Access Authority XY

**Contact(s)** XY

**Access Conditions** Data freely available under CC BY ND

### Citation Requirements

Files Description

Dataset contains 1 file(s)

## Covid19 Mobility and Borders

### File Content

**Cases Variable(s) Producer**

**Version of the microdata**

**Notes**

The dataset correlates policies related to border control and policies regulating domestic mobility in 27 countries in Europe. It provides information on the type of border control (open border, mostly open border, mostly closed border, closed border), the policies related to human movement inside the states' borders (no restrictions; population invited to stay at home, schools closed; businesses and schools closed; curfews), the date of announcement, and the date of entry into force. The interactive visualization can be accessed through this link: <https:// tabsoft.co/2YqC5is>

373

8

Tabular

CSV file containing one line per each policy related to border control and policies regulating domestic mobility

# Variables Groups

Dataset contains 0 groups

# Variables List

Dataset contains 8 variable(s)

## File Covid19 Mobility and Borders

|  | **Name** | **Label** | **Data Type** | **Valid** |
| --- | --- | --- | --- | --- |
|  |  |  |  |  |
| 1 | **Type** | Type | discrete | 373 |
| 2 | **Limitation** | Type of restriction (international travel/domestic mobility) | discrete | 373 |
| 3 | **Country** | Country | discrete | 373 |
| 4 | **Expert** | Country expert | discrete | 373 |
| 5 | **Dateofdecision** | Date of decision | discrete | 373 |
| 6 | **Dateofimplementation** | Date of implementation | discrete | 373 |
| 7 | **Details** | Details of the restriction | discrete | 373 |
| 8 | **Source** | Source | discrete | 347 |

Alphabetical List

Dataset contains 8 variable(s)

**n**

| **Name** | **Label** | **Page** |
| --- | --- | --- |
|  |  |  |
| **Country** | Country | **8** |
| **Dateofdecision** | Date of decision | **9** |
| **Dateofimplementatio** | Date of implementation | **9** |
| **Details** | Details of the restriction | **9** |
| **Expert** | Country expert | **9** |
| **Limitation** | Type of restriction (international travel/domestic mobility) | **7** |
| **Source** | Source | **9** |
| **Type** | Type | **7** |

# Variables Description

Dataset contains 8 variable(s)

Comment: these figures indicate the number of cases found in the data file. They cannot be interpreted as summary statistics of the population of interest.

## File Covid19 Mobility and Borders

1 **Type** Type

**Information** Data Type: discrete, Format: numeric, Range: 0-2, Missing: *

**Statistics** Valid=373, Invalid=0

**Definition** Indicates if the current record describes a restriction that affects mobility within national borders (1) or mobility from/towards other countries (2)

1

234 b

62.7%

s

37.3%

139

2

N b Percentage

Value

La

el

Mo rest

ility rictions

Bor clo

ders ure

Comment: these figures indicate the number of cases found in the data file. They cannot be interpreted as summary statistics of the population of interest.

2 **Limitation** Type of restriction (international travel/domestic mobility)

**Information** Data Type: discrete, Format: numeric, Range: 0-3, Missing: *

**Statistics** Valid=373, Invalid=0

**Definition** Indicates the degree of severity of the restriction described in the current record.

Values of limitations range from 0 to 3, interpretation depends on the value of the variable "Type".

Togther with the variable "Type", the "Limitation" defines an international or domestic restriction with the following key:

Type-Limitation-Restriction 1 - 0 - No restrictions

1 - 1 - Population invited to stay at home, schools mostly closed but with exceptions - Moderate

1 - 2 - Population invited to stay at home, some businesses and schools closed, and light sanctions for those who transgress - Moderate with sanctions

1. - 3 - Population invited to stay at home, most businesses and all schools closed, curfews, and heavy enforcement of these measures - Strict confinement
2. - 0 - Open border - Open border

2 - 1 - Mostly open border, with additional controls at the entry - Border open, some restrictions 2 - 2 - Border closed with generous exceptions - Border closed, many exceptions

2 - 3 - Border closed with almost no exceptions - Border closed

La

el

non

e

min

imal

| Value N b Percentage | | | | | | |
| --- | --- | --- | --- | --- | --- | --- |
| 0 | 11 |  | 2.9% |  |  |  |
| 1 | 116 |  |  |  | 31.1% |  |
| 2 | 181 d |  |  |  |  | 48.5% |
| 3 | 65 x |  |  | 17.4% |  |  |

Comment: these figures indicate the number of cases found in the data file. They cannot be interpreted as summary statistics of the population of interest.

mo

erate

ma

imal

## File Covid19 Mobility and Borders

3 **Country** Country

La

el

N b Percentage

Norway Romania Slovenia Spain Sweden Switzerland

United Kingdom

24

10

6

20

11

22

7

6.4%

2.7%

1.6%

5.4%

2.9%

5.9%

1.9%

| Austria | 29 |  |  |  |  | 7.8% |
| --- | --- | --- | --- | --- | --- | --- |
| Belarus | 6 |  | 1.6% |  |  |  |
| Belgium | 10 |  |  | 2.7% |  |  |
| Cyprus | 20 |  |  |  | 5.4% |  |
| Denmark | 23 |  |  |  | 6.2% |  |
| Estonia | 6 |  | 1.6% |  |  |  |

| **Information** | Data Type: discrete, Format: Character, Missing: * |
| --- | --- |
| **Statistics** | Valid=373, Invalid=0 |
| **Definition** | Country issuing restrictions |
| Value |  |

| Finland | 24 |  |  |  | 6.4% |
| --- | --- | --- | --- | --- | --- |
| France | 10 |  | 2.7% |  |  |
| Germany | 18 |  |  | 4.8% |  |
| Greece | 12 |  | 3.2% |  |  |
| Hungary | 20 |  |  | 5.4% |  |
| Iceland | 12 |  | 3.2% |  |  |
| Ireland | 6 | 1.6% |  |  |  |

| Italy | 13 3.5% | | | |
| --- | --- | --- | --- | --- |
| Latvia | 7 | 1.9% |  |  |
| Liechtenstein | 14 |  | 3.8% |  |
| Lithuania | 14 |  | 3.8% |  |
| Luxembourg | 18 |  |  | 4.8% |
| Malta | 11 2.9% | | | |

Comment: these figures indicate the number of cases found in the data file. They cannot be interpreted as summary statistics of the population of interest.

## File Covid19 Mobility and Borders

4 **Expert** Country expert

**Information** Data Type: discrete, Format: numeric, Missing: *

**Statistics** Valid=373, Invalid=0

**Definition** Investigator in charge of the country analysis

La

el

Value 1

2

3

4

5

N b Percentage 63

29

71

16.9%

7.8%

19.0%

15

100

4.0%

26.8%

| 6 | 23 | 6.2% |  |
| --- | --- | --- | --- |
| 7 | 18 | 4.8% |  |
| 8 | 54 |  | 14.5% |

Comment: these figures indicate the number of cases found in the data file. They cannot be interpreted as summary statistics of the population of interest.

5 **Dateofdecision** Date of decision

**Information** Data Type: discrete, Format: Character, Missing: *

**Statistics** Valid=373

**Definition** Date when the restriction was publicly announced

*Frequency table not shown (78 Modalities)*

6 **Dateofimplementation** Date of implementation

**Information** Data Type: discrete, Format: Character, Missing: *

**Statistics** Valid=373

**Definition** Date when the restriction was implemented

*Frequency table not shown (83 Modalities)*

7 **Details** Details of the restriction

**Information** Data Type: discrete, Format: Character, Missing: *

**Statistics** Valid=373

**Definition** Explanation of what the restriction entails

8 **Source** Source

**Information** Data Type: discrete, Format: Character, Missing: *

**Statistics** Valid=347

**Definition** Reference source for the restriction

# Appendices

## The documents listed below are available on the nccr - on the move website (nccr- onthemove.ch)

Other resources

*<https://public.tableau.com/profile/nccr.on.the.move*

**Website**
